# An Account on BiVO_4_ as Photocatalytic Active Matter

**DOI:** 10.1021/accountsmr.3c00021

**Published:** 2024-03-15

**Authors:** Sandra Heckel, Martin Wittmann, Marc Reid, Katherine Villa, Juliane Simmchen

**Affiliations:** † Physical Chemistry, TU Dresden, Zellescher Weg 19, 01069 Dresden, Germany; ‡ Department of Pure and Applied Chemistry, University of Strathclyde, 295 Cathedral Street, Glasgow G1 1XL, United Kingdom; ¶ Institute of Chemical Research of Catalonia (ICIQ), The Barcelona Institute of Science and Technology (BIST), Av. Països Catalans, 16, 43007 Tarragona, Spain

## Abstract

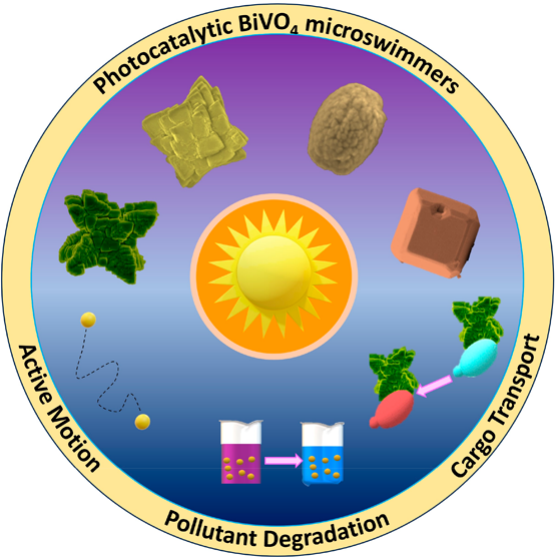

Photocatalytic materials are gaining popularity and research investment for developing light-driven micromotors. While most of the early work used highly stable TiO_2_ as a material to construct micromotors, mostly in combination with noble metals, other semiconductors offer a wider range of properties, including independence from high-energy UV light. This review focuses on our work with BiVO_4_ which has shown promise due to its small band gap and resulting ability to absorb blue light. Additionally, this salt's well-defined crystal structures lead to exploitable charge separation on different crystal facets, providing sufficient asymmetry to cause active propulsion. These properties have given rise to fascinating physical and chemical behaviors that show how rich and variable active matter can become. Here, we present the synthesis of different BiVO_4_ microparticles and their material properties that make them excellent candidates as active micromotors. A critical factor in understanding inherently asymmetric micromotors is knowledge of their flow fields. However, due to their small size and the need to use even smaller tracer particles to avoid perturbing the flow field, measuring flow fields at the microscale is a difficult task. We also present these first results, which allow us to demonstrate the correlation between chemical reactivity and the flow generated, leading to active motion. Due to the nontoxic nature of BiVO_4_, these visible-light-responsive microswimmers have been used to study the first steps toward applications, even in sensitive areas such as food technology. Although these initial tests are far from being realized, we have to face the fact that a single microswimmer will not be able to perform macroscale tasks. Therefore, we present the reader with the first simple studies of collective motion, hoping for many new contributions to the field. The one-step synthesis of BiVO_4_ clearly paves the way for studies requiring large numbers of particles. We predict that the combination of promising applications for a nontoxic material which is readily synthesized in large quantities will contribute pivotally to advance the field of active matter beyond the proof-of-concept stage.

## 1 Introduction

### 1.1 Light-Driven Micromotors

Visible light refers to a fraction of electromagnetic radiation that is perceived by the human eye, providing the raw data that, in turn, enables vision. On earth, sunlight is the main source of energy. Nature takes advantage of it through different mechanisms that transform the energy stored in electromagnetic radiation into chemical energy or mechanical (kinetic) processes. Because the energy content of light is easily variable by employing different wavelengths and intensities, light has proven to be an attractive energy resource to power-up and activate synthetic active colloids. Remote energy transmission and optical actuation have become a frequently used tool in many different approaches.^1^ There are excellent reviews explaining the history of light driven active matter in-depth and under different perspectives,^2,3^ for which we will only summarize general information and focus on the contemporary developments on BiVO_4_ here.

Since photoactivated colloids require light to excite chemical processes that generate the self-created chemical gradients, their motion can easily be activated by switching the light source on and off. One of the earliest photocatalytic microswimmer examples are Au@TiO_2_ Janus particles.^4,5^ UV light illumination causes the generation of electron–hole pairs in TiO_2_, which are then separated by a Schottky junction, leading to electron transfer to the Au hemisphere. In short, the photoactivation of the photocatalytic material enables the catalytic decomposition of a fuel that forms a gradient which propels the swimmers, whereas in absence of irradiation, they simply perform Brownian motion.^5^ The control-enabling advantages of light over other energy sources have led to an increase in related research on microswimmer technologies. Soon, further materials besides TiO_2_ and ZnO were developed that allow to switch to visible light. Possible pathways to visible-light-activated photocatalysts are, for example, the engineering of heterostructures or switching to semiconductors with a smaller bandgap.

### 1.2 Properties of Bismuth Vanadate

Vanadates have historically been used as a variety of pigments, or as test reagents for detecting illicit substances.^6^ As a mixed-metal oxide, BiVO_4_ has recently gained interest as catalyst for photoelectrochemical water splitting, as it exhibits favorable valence and conduction band potentials for water oxidation.^7^ Extensive research on the fabrication of BiVO_4_ photoanodes for photoelectrochemical processes has been conducted over the past 20 years. In particular, the monoclinic crystalline structure is more appealing for photocatalytic processes, due to its short band gap (2.4 eV).^8^

The band gap type of BiVO_4_ has been a topic of discussion: Zhao et al. found, in calculations, that the band gap between conduction band minimum and valence band maximum of BiVO_4_ is indirect, but direct band gaps with slightly higher energies are also present.^9^ Walsh et al. confirmed the dominance of the direct band gap^10^ by good linear correlations between (*αhν*)^2^ and *hν* in the Tauc plot of most experimental works.^11^

The theoretically achievable solar to hydrogen (STH) efficiency of BiVO_4_ in water splitting is promising for future applications.^12^ However, the practically achieved STH remains at lower values to date, owing to the short carrier diffusion length and the slow hole transfer at the BiVO_4_/electrolyte interface.^13^

What makes BiVO_4_ particles so relevant for microswimmer systems is the efficient spatial electron–hole separation upon illumination without the need of a cocatalyst.^14^ Monoclinic BiVO_4_ crystallizes in an octahedral truncated bipyramidal shape, with mainly {010} and {110} facets being exposed. In 2013, Li et al. showed experimentally that photogenerated electrons are drawn to the {010} facet and holes to the {110} facet by photoreducing and -oxidizing a cocatalyst onto the different facets, and that the amount of oxygen evolution from water oxidation increased by a factor of 160 when particles with photodeposited Pt and Co_3_O_4_ were compared to plain BiVO_4_ particles, compared to a factor of 9 for unselectiveley impregnated particles.^15^

In the field of light-driven micro/nanoswimmers, BiVO_4_-based systems are becoming ideal candidates to develop noble-metal-free micromachines without requiring complex fabrication techniques. Depending on their morphology, these devices have shown interesting swimming behaviors as a result of the photogenerated fluid flows, interactions with the substrate, and/or presence of external entities. In the following sections, we will describe our views on crucial points of motion mechanisms and interactions and give an outlook on future applications.

## 2 Synthesis of Defined BiVO_4_ Microparticles

Syntheses of bismuth oxides are typically carried out in the acidic regime to solubilize bismuth ions, which otherwise react with water to form the insoluble salt bismuth oxynitrate (eq 1). This can be achieved using a variety of acids (eq 2), frequently HNO_3_.
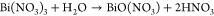
1
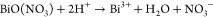
2In the synthesis, the vanadate precursor NH_4_VO_3_ first forms vanadic acid (H_3_VO_4_), which eventually reacts with bismuth ions to form the insoluble mixed oxide BiVO_4_. These particles can be either single-crystals or of polycrystalline nature, leading to different properties and advantages to either outcome. Within the hydrothermal synthesis approach, tuning the ratio of reagents, adapting pH values of the solutions,^16^ varying additives, or even the reaction medium, results in different morphologies.^17^

While other structures are available, we summarize typical syntheses that have been used to produce colloidal homogeneous BiVO_4_ micromotors and their unique properties.

### 2.1 Polycrystalline Microparticles

**Figure 1:**
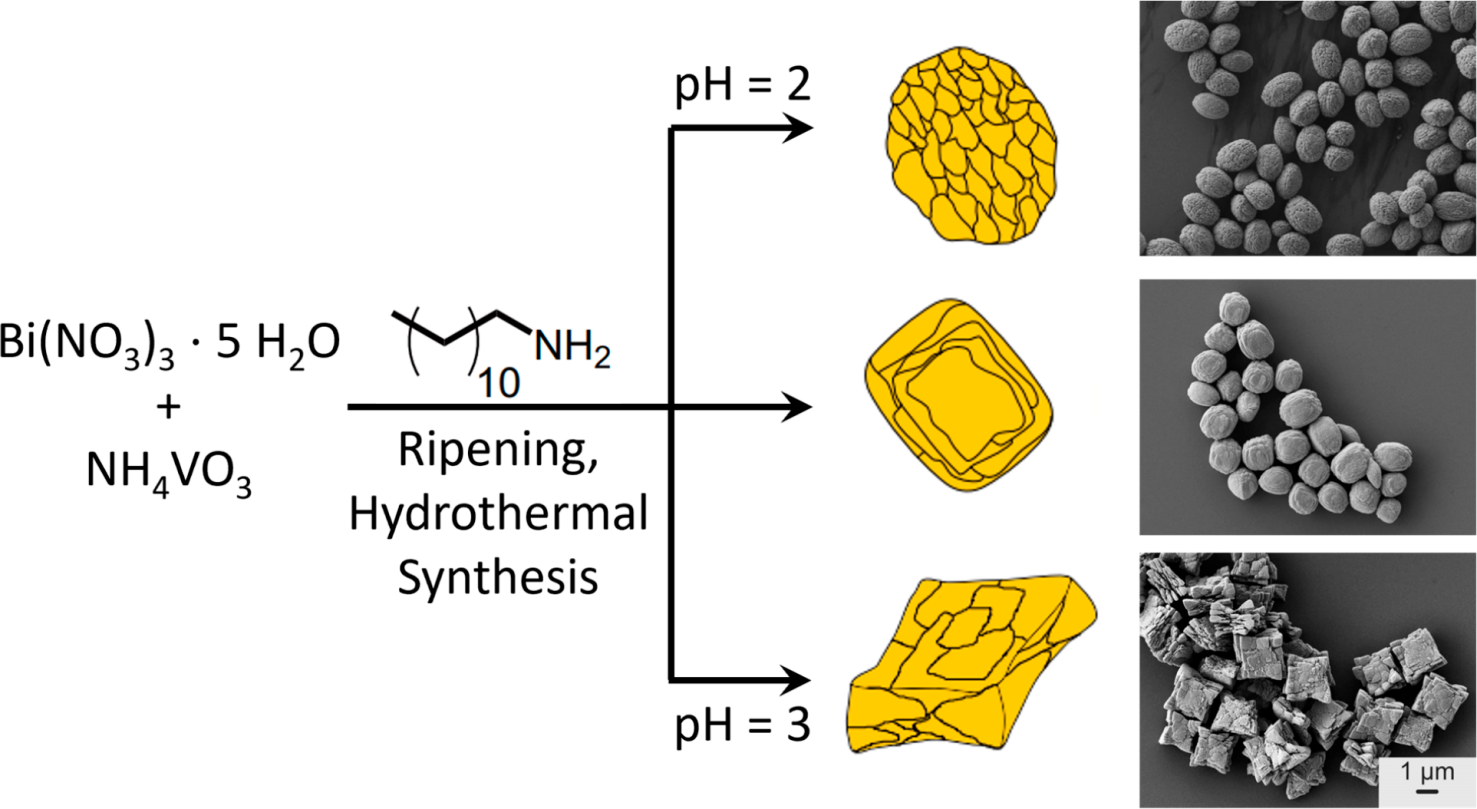
Synthesis scheme and SEM images of polycrystalline BiVO_4_ microparticles at pH 2–3, causing the formation of particles with spheroidal and rectangular and stacked morphologies. Adapted with permission from ref (16, 18). Copyright 2021 MDPI.

In a typical synthesis of polycrystalline BiVO_4_ colloids, the precursors Bi(NO_3_)_3_ and NH_4_VO_3_ are dissolved in a solvent mixture of ethanol, ethylene glycol (EG) and HNO_3_, where EG executes two functions: it serves as a solvent that stabilizes BiO^+^ ions and thereby increases the solubility of Bi(NO_3_)_3_ and acts as surfactant, stabilizing the {010} facet of the particles.^19^ At pH = 2, spheroidal particles were obtained, while if the pH was increased, the shape of the particles became rather rectangular, and at pH = 3, a cuboidal, almost star-shaped morphology is observed (Figure 1). In the presence of the chelating agent ethylenediaminetetraacetic acid more defined shapes, as well as single and double star-shaped particles are obtained, where the irregularly shaped, thin crystallites are stacked on top of each other, which leads to visible clefts between the layers.^20,21^

The quite obvious change in particle shape over a rather narrow pH range from 2 to 3 can be explained by the concentration of H^+^ ions, which influences crystal growth through the solubilization of bismuth ions.

Among the first reports using BiVO_4_ as active matter, particles based on polycrystalline geometries were used that resemble stars or shuriken.^20,21^ These showed the peculiarity of standing up on irradiation and swimming in an upright position.

### 2.2 Single-Crystals

**Figure 2:**
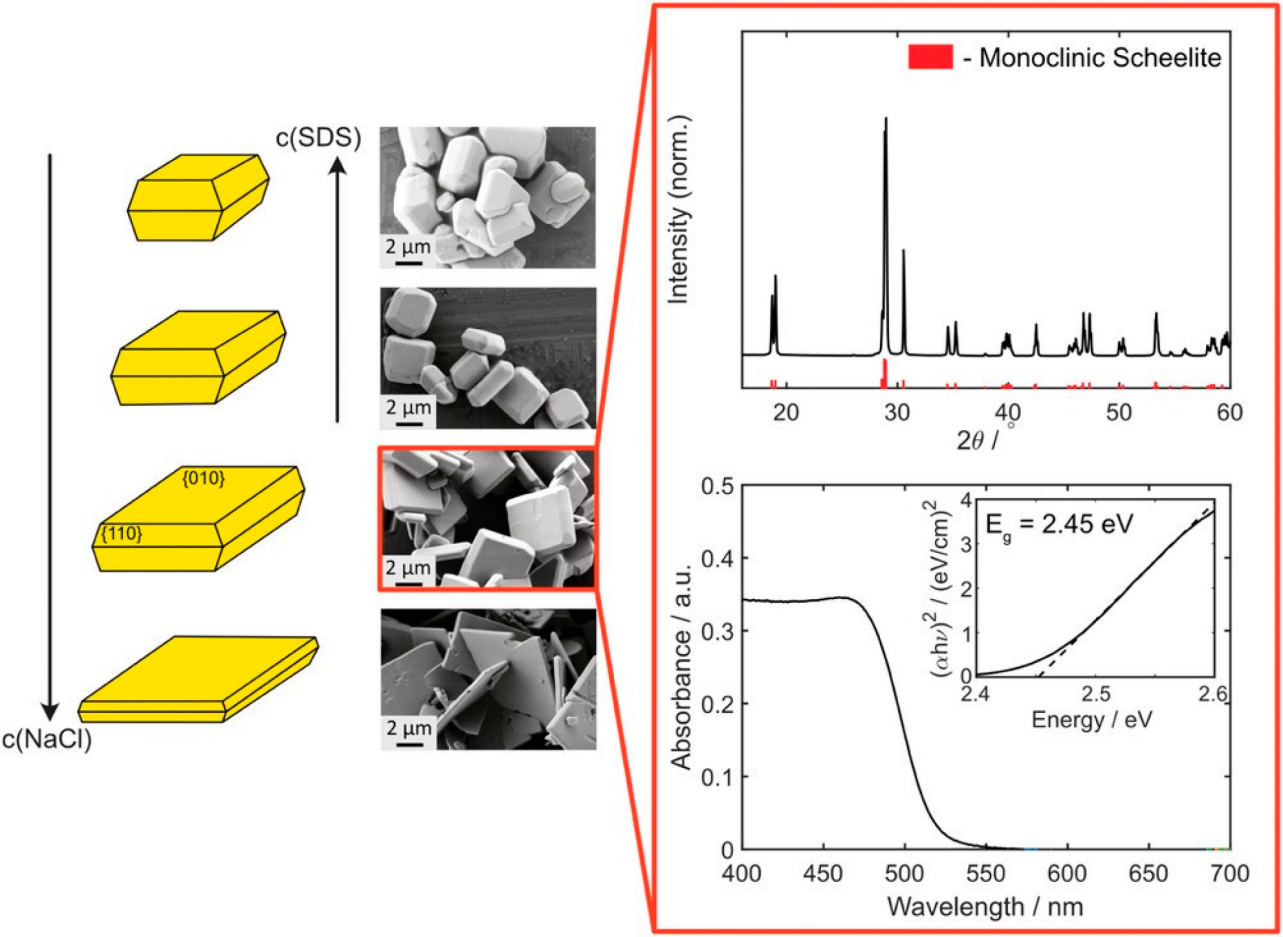
Left: Scheme showing the impact of addition of NaCl and SDS marked by arrows on crystal morphology and SEM images of BiVO_4_ particles synthesized at different concentrations of NaCl and SDS: top: 0.05 M NaCl and 0.05 M SDS; second: 0.05 M NaCl and 0.005 M SDS; third: 0.05 M NaCl; bottom: 1.0 M NaCl. Right: XRD pattern and UV–vis absorption plot of the selected BiVO_4_ sample. The monoclinic scheelite crystal structure can be clearly identified (Reference: PDF-Nr. 16-688, red). In the absorption spectrum, which was recorded with a dilute particle solution in water, it can be seen that light in the UV range up to the visible range is absorbed. Calculation of the band gap via the Tauc plot for a direct semiconductorgives a value of *E*_g_ = 2.45 eV. Reproduced with permission from ref (22). Copyright 2022 Wiley Online Library.

Besides polycrystalline particles, single-crystals are also promising candidates for developing efficient light-driven microswimmers. This is due to a high electron–hole separation rate under illumination, resulting from the absence of grain boundaries. Shape and uniformity of single-crystalline BiVO_4_ microparticles can be strongly improved by the addition of surfactants during the synthesis. An adapted protocol including the addition of NaCl and SDS (sodium dodecyl sulfate) as capping agents allowed the tuning of aspect ratios and yielded in rather homogeneous morphologies.^22^ Similar to the polycrystalline particles, XRD and DRS analysis revealed the monoclinic BiVO_4_ structure and a band gap of 2.45 eV for the single crystals (Figure 2).

## 3 Assessment of Electron–Hole Separation of the Microswimmers Based on Their Morphology

**Figure 3:**
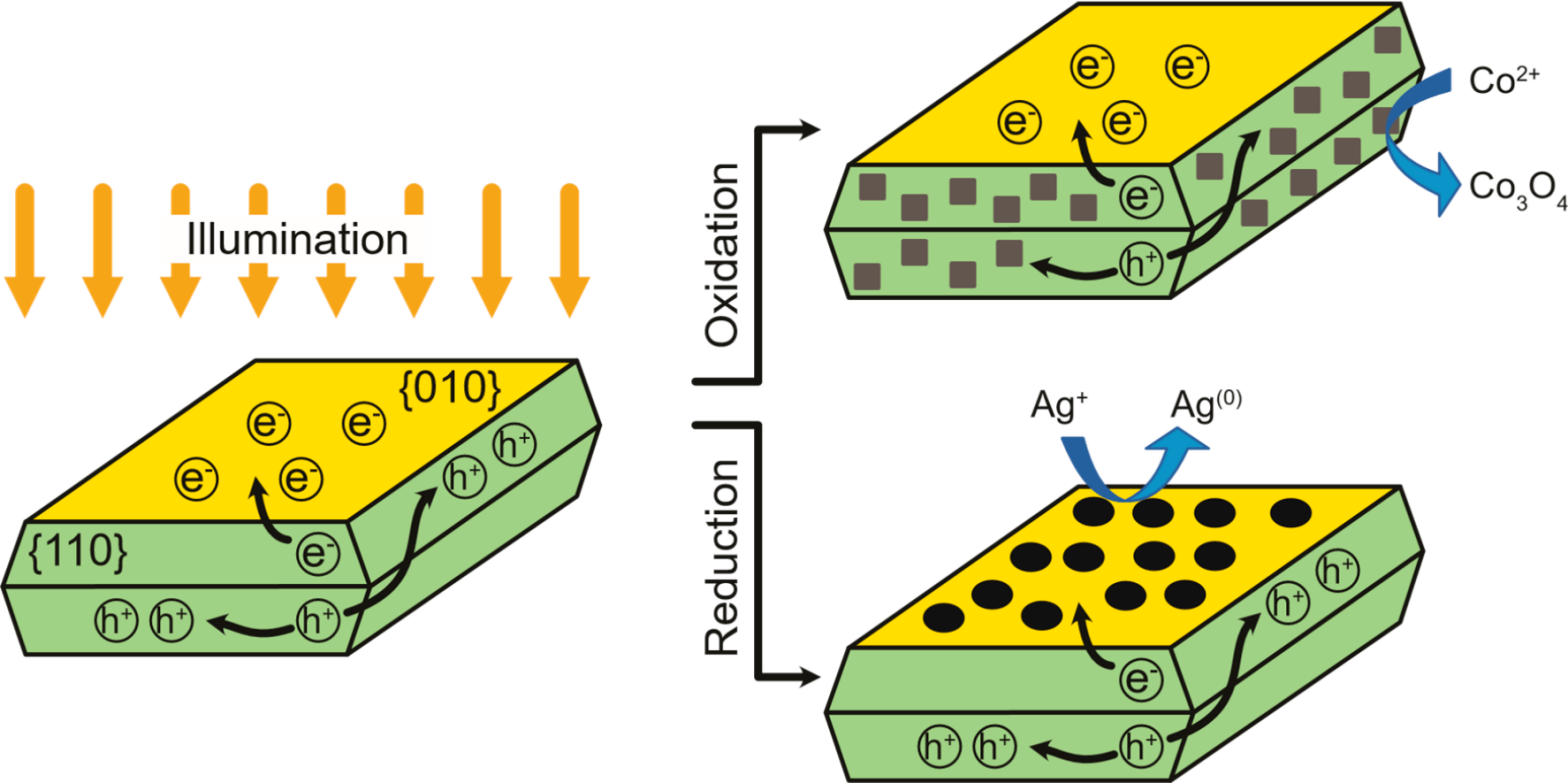
Scheme of charge separation on facets of BiVO_4_ and identification by photodeposition. Reductive areas can be identified by reduction of noble metal ions like Ag^+^ to Ag, while Co_3_O_4_ is preferentially deposited on oxidative areas. Reproduced with permission from ref (22). Copyright 2022 Wiley Online Library.

To investigate if this preferential crystal orientation along with the surface heterojunction commonly observed for BiVO_4_ crystals between their {010} and {110} facets can serve as a means to separate excited charge carriers on the particle surface, photooxidation and reduction experiments were carried out with the different particle geometries. The principle of these experiments is shown in Figure 3.

**Figure 4:**
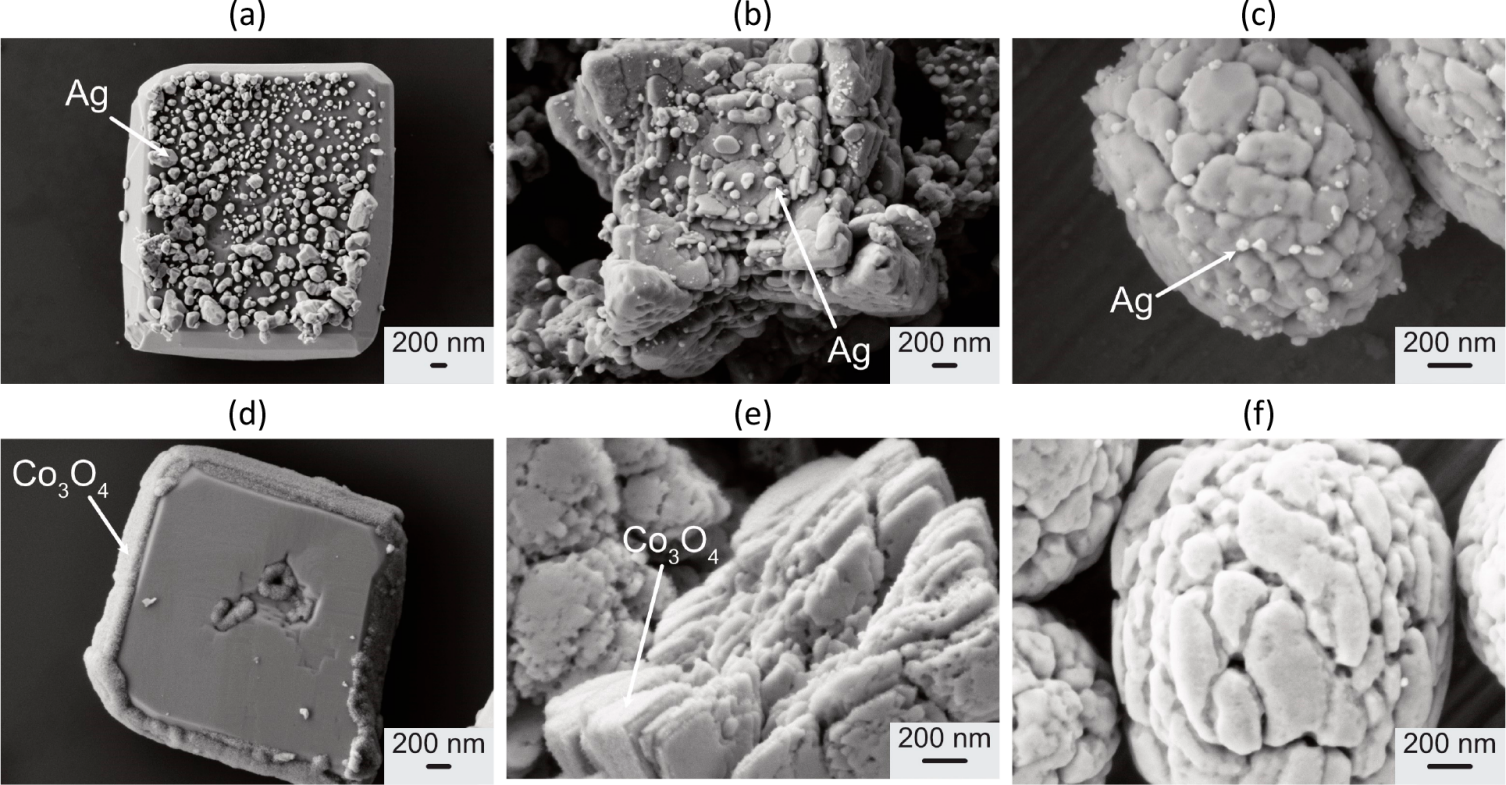
SEM images of (a, d) single-crystalline, (b, e) stacked, and (c, f) spheroidal BiVO_4_ particles after photodeposition with (a–c) AgNO_3_ and (d–f) CoCl_2_ and NaIO_3_. Reproduced with permission from refs (18, 22). Copyright 2022 Wiley Online Library.

To identify electron-rich facets using photodeposition, aqueous dispersions of BiVO_4_ samples were illuminated with a halogen lamp while in the presence of Ag^+^ ions, so that Ag^+^ ions are reduced to Ag^(0)^ by photogenerated electrons (eq 3).^14^ No hole scavenger was required, indicating that H_2_O was oxidized to O_2_ or ^•^OH (eqs 4 and 5) on the side faces of the particles.
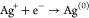
3
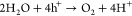
4
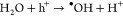
5SEM images of the selective photoreduction on different particle geometries are shown in Figure 4a–c. Given that the electrons preferably accumulate on the {010} facets at the top and bottom of the particles, these charges lead to localized deposition of noble materials. This behavior is consistent over the 3 different geometries but more pronounced for singlecrystalline BiVO_4_ particles. For stacked particles deposition is mainly observed on the top and bottom areas, but also with less intensity into the clefts between the stacked layers. To confirm the presence of hole-rich facets, BiVO_4_ particles have been illuminated in the presence of Co^2+^ ions, which can be oxidized to Co_3_O_4_ by photogenerated holes.^15^ As an electron scavenger, NaIO_3_ was added to the solution. Under illumination, IO_3_^–^ is reduced to I^–^.
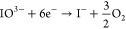
6
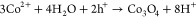
7

As observed before, the deposition on single-crystalline particles on the {110} facets is much more pronounced compared to stacked particles. For spheroidal particles no photodeposited Co_3_O_4_ was detected (see Figure 4d–f).

To summarize, a clear orientation of the crystal structure is visible in single-crystalline microparticles, leading to preferentially exposed {010} facets and charge carrier separation upon illumination.

## 4 Characterization of Motion

When aqueous dispersions of microparticles are mixed with H_2_O_2_ and illuminated with visible light, excited charge-carriers are created within the BiVO_4_ microparticles which leads to respective reduction and oxidation processes of H_2_O_2_. This duality results in the photogeneration of different ions and chemical species that diffuse at different rates, creating an asymmetrical chemical gradient around the particles that triggers their ballistic motion by phoretic mechanisms.

As the degree of asymmetry is mainly determined by charge carrier separation by the surface heterojunction between {010} and {110} facets in BiVO_4_, different degrees of ballistic motion can also be expected for the three particle morphologies.

**Figure 5:**
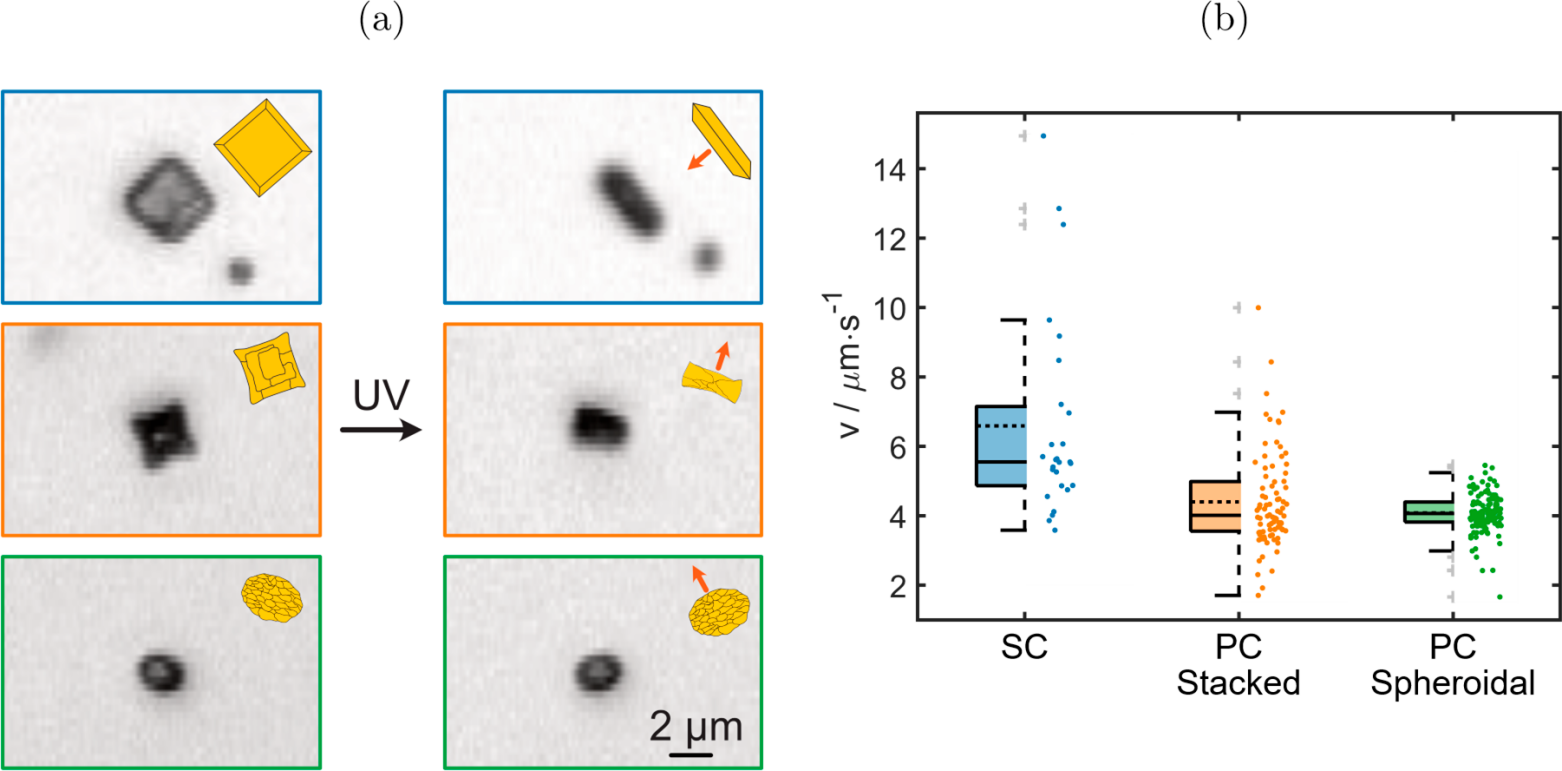
(a) Orientation of single-crystalline, stacked, and spheroidal particles in a 0.1 wt % aqueous H_2_O_2_ solution in dark conditions and under irradiation with (385 nm) UV light. UV light irradiation causes a reorientation into an upright position for the single-crystalline and stacked particles, which was not observed for spheroidal particles. (b) Box plots of average instantaneous speeds of at least 25 single-crystalline (SC), stacked (PC stacked), and spheroidal (PC spheroidal) particles in a 0.1 wt % aqueous H_2_O_2_ solution under (385 nm) UV light irradiation. Summarized behaviors from refs (16, 18, 22, 23).

It is well-known that many Janus particle microswimmers swim in a fixed 90° angle to the substrate because the self-induced flows around them lead to a reorientation.^24,25^ Similarly, we observe a reorientation for BiVO_4_: in absence of suitable radiation, particles lie horizontally on their bottom face, while light irradiation causes a 90° rotation into an upright position, where the particles stand on one of their side faces (see Figure 5a).^2,21,22^ We believe that analogous to spherical swimmers, a combination of hydrodynamics, chemical fields, and second order effects are causing this behavior. For the spheroidal microparticles, observation of particle rotation around their long axis is nearly impossible in an optical microscope with two-dimensional resolution. Yet, all particles show increased activity and move in a certain direction, which is indicated by red arrows.

Comparing individual speeds, the single-crystalline BiVO_4_ microparticles are fastest with a mean speed of 6.59 μm s^–1^, while polycrystalline particles are characterized by lower mean speeds (Figure 5b). Both, the stacked and spheroidal particles swim at speeds just over 4 μm s^–1^ in the ballistic regime, as verified looking at the slope of the bilogarithmic MSD plot for the time up to τ = 1 s.

### 4.1 Flow Fields

Microscale motion is caused by interactions between the microswimmer surface, the solutes, and the substrate on which they are moving. These interactions cause fluid flows in the vicinity of the microswimmer, which eventually lead to the fluid executing forces on the microswimmer and the microswimmer back on the fluid, resulting in overall force- and torque-free motion.

Fluid motion on the microscale is described by the Navier–Stokes equations, with three basic solutions yielding possible flow fields. A stokeslet or force monopole is the simplest solution obtained for a particle driven by an external point force. A force dipole is the simplest solution of the Stokes equations that can describe active, self-induced motion and respects the boundary condition that the net force acting on an active particle is zero. Therein, two forces with the same magnitude point in opposite directions, and we can differentiate between two distinct cases: for outward pointing forces, the flow field is extensile and the resulting microswimmer is denominated pusher. In the opposite case, the forces point inward, forming a contractile flow field and the swimmer is called a puller.^26^

Experimentally, the flow field of microswimmers can be obtained by observing the trajectories of small passive tracer particles. Depending on the specific details, the measurements are referred to as particle image or tracking velocimetry, PIV or PTV, respectively, giving insights how the self-created solutes interact with the swimmer’s surface, resulting in distinct flow profiles.^27^ Due to the experimental difficulties on the microscale, the use of particle velocimetry on micromotors has been very limited and not yet extended beyond spherical microswimmers^28^

For BiVO_4_ microswimmers, these techniques were difficult to adapt, which was not only due to the deviation from spherical shapes for single-crystalline and stacked polycrystalline morphologies but also the origin of the activity is caused by photochemical reactions, leading to a completely different source distribution.

To reliably track the flows, the choice of tracer particles is of the highest importance. Ideally, the tracer size should not be larger than 1*/*50 of the object diameter and the materials should not engage in chemical reactions. Au nanoparticles (Au NPs) proved to be a good material choice for BiVO_4_ due to their high density (confining them mostly in one plane) and their excellent contrast in light microscopy. Despite their low mean diameter of (250 ± 22)nm, individual particles could be tracked in optical microscopy. Au NPs of that size also did not show significant catalytic activity for H_2_O_2_ decomposition.

**Figure 6:**
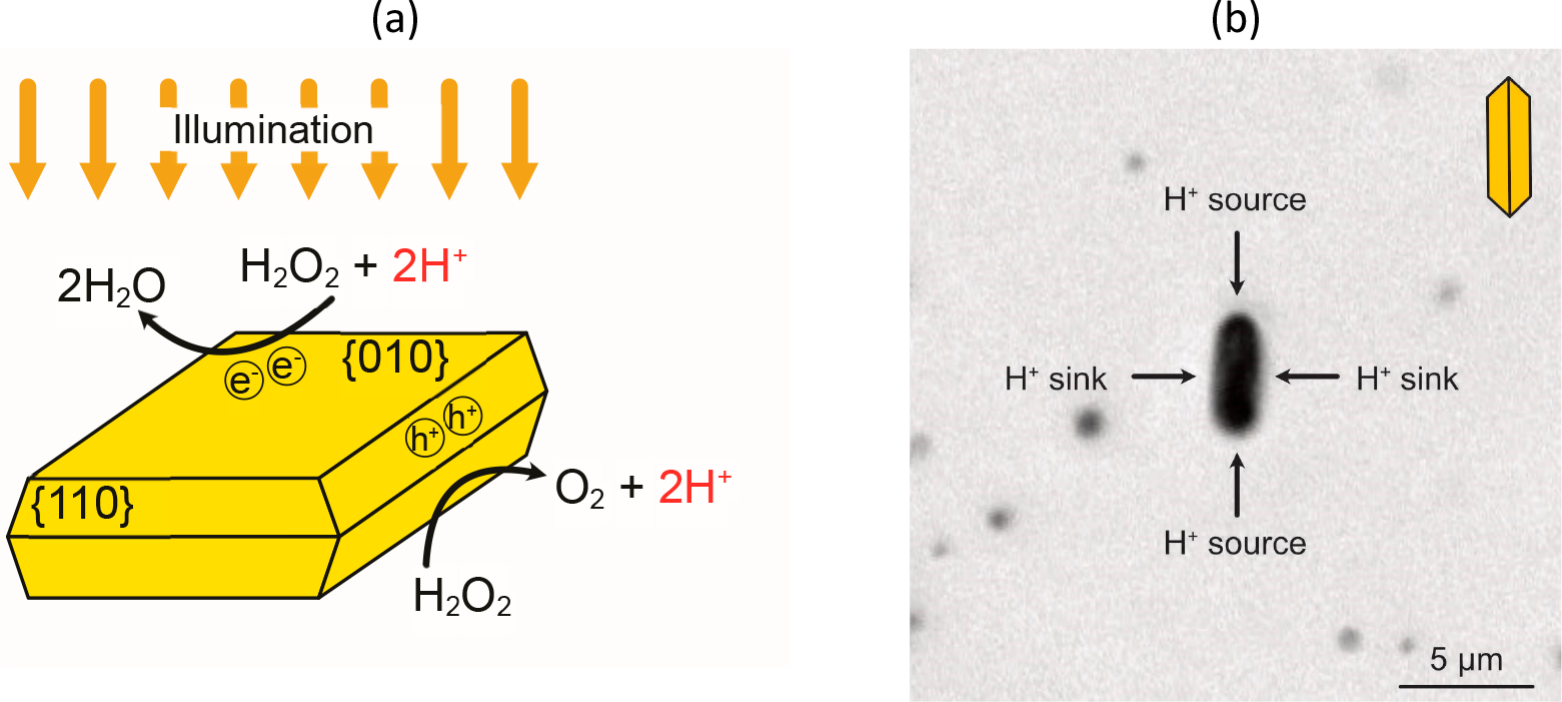
(a) Schematic illustration of separated half reactions of H_2_O_2_ decomposition on the facets of single-crystalline BiVO_4_ particles. H_2_O_2_ reduction, which consumes H^+^ ions preferably takes place on the {010} facets, while protons are produced in the H_2_O_2_ oxidation on the {110} facets. (b) Light microscopy image of a typical flow field experiment. Reproduced with permission from refs (18, 22). Copyright 2022 Wiley Online Library.

The strong separation of oxidation and reduction sites in the single-crystalline BiVO_4_ microparticles suggests that their motion pattern is dominated by self-electrophoresis, for more details see reference.^18^ Under illumination, H^+^ ions are produced at the {110} facets and consumed in a reduction reaction at the {010} facets (Figure 6a). Consequently, fluid flows from the side to the top and bottom faces of the microswimmer would be expected. In contrast to conventional self-electrophoretic microswimmers, the single-crystalline BiVO_4_ colloids should therefore give rise to a source quadrupole in the focus plane due to their geometry: When in an upright position, two sides with {110} facets are illuminated by light and act as proton sources, while the two {010} facets consume protons (see Figure 6b).

**Figure 7:**
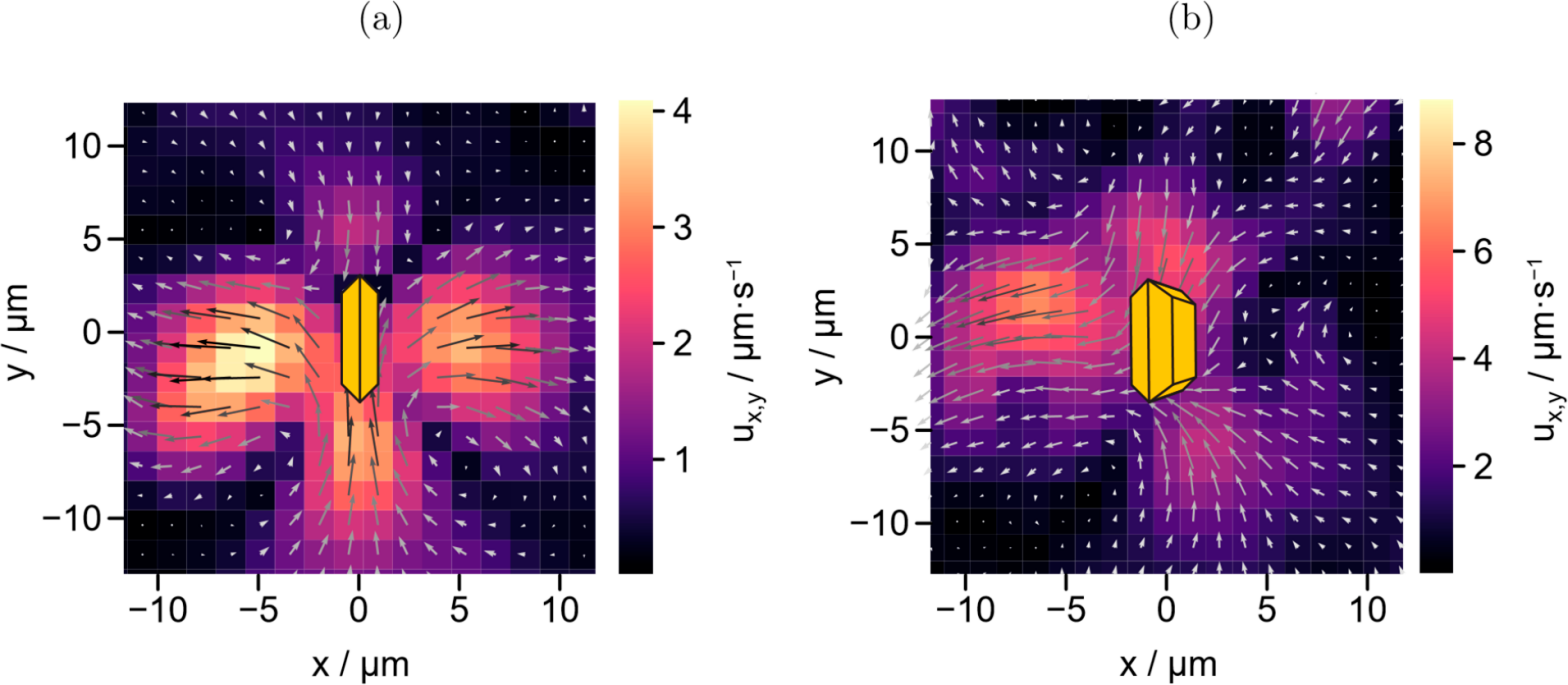
Exemplary flow fields around attached (a) upright and (b) inclined microswimmers. Orientation and position of the swimmers are indicated by the yellow particle sketch. In the surrounding fluid of both colloids, flows from oxidative to reductive facets can be observed. Reproduced with permission from ref (22). Copyright 2022 Wiley Online Library.

Obtained flow fields are depicted in Figure 7a and b, comparing particles in an upright position (i.e., a 90° angle between the microswimmers and glass substrates) and an inclined position, revealing that tracer velocities and motion direction vary strongly. Still, common trends include the inward flows from the long side {110} toward the top and bottom {010} facets, from where the fluid appears to be pushed outward. The source quadrupole which is caused by electron driven reactions on the {010} facets (along the *x*-axis) and hole-induced reactions on faces with {110} facets (along the *y* axis), is finally overlaid with hydrodynamic constraints and results in a flow field with similarities to a pusher-type force dipole. The asymmetry required for active motion is most likely ascribed to an overlap of intrinsic surface asymmetries and self-shadowing, which then translates into different amounts of created charge-carriers and subsequently into a more pronounced product gradient. These effects could be self-enhancing, which is underlined by the observation, that inclined microswimmers are often seen to move along rather straight trajectories.

Preliminary tests showed that flows caused by polycrystalline BiVO_4_ microparticles were too small to be resolvable with the PTV methodology described.

### 4.2 Motion Mechanisms

Building on the charge carriers produced by photophysical interactions of irradiation with BiVO_4_, it is reasonable to assume the motion mechanism in H_2_O_2_ to be electrophoretic. From a mechanistic and kinetic perspective, H_2_O_2_ degradation is a complex reaction: Under acidic and neutral conditions on the surface of a suitable catalyst, hydrogen peroxide disproportionates into H_2_O and O_2_ (eq 8).
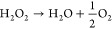
8When charge carriers are available in the system, the disproportionation reaction can also be separated into an oxygen-forming oxidation half-reaction and a reducing half-reaction of H_2_O_2_ (eqs 9 and 10).


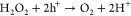
9
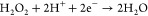
10Looking at the equations above, it becomes apparent that two different species can create chemical gradients, leading to phoretic motion by different mechanisms: O_2_ resulting in neutral self-diffusiophoresis, and H^+^, which also generates an electric field and therefore results in self-electrophoresis.^29,30^ While simple neutral diffusiophoretic models capture certain phenomena, many experimentally observed behaviors cannot be explained considering only neutral reaction products.^31^ Ionic diffusiophoresis considering also an endogeneous electric field, resulting from the diffusion of charged species, broadens the applicability.^32^ Electrophoretic micromotion was first described for bimetallic Au–Pt rods in dilute H_2_O_2_.^33,34^

The overall catalytic reaction is split into reduction and oxidation half reactions (see also eqs 9 and 10), happening at opposite sides of the swimmer body, with electrons migrating through it, causing a net displacement in a self-generated electric field, driven by the motion of ions and subsequent flows in a thin layer around the particle.^29,35^ In analogy to TiO_2_ based microswimmers^30^ we assume the dominant mechanism to be electrophoretic. This is underpined by the change in direction of particle motion upon an inversion of the zeta potential as well as further environmental parameters such as the pH and conductivity.

### 4.3 Environmental Factors Affecting Motion

#### Conductivity

The conductivity of the solution is a crucial parameter to prove ionic or electrophoretic influences,^31,36^ since with increasing conductivity, the endogenous electric field around a microswimmer decreases according to Ohm’s law. For self-electrophoresis, the swimmer speed is predicted to be proportional to this self-produced electric field,^35^ while for self-diffusiophoresis the relations are much less clear, as factors like viscosity and catalytic activity are influenced by the ion concentration. A test for stacked BiVO_4_ particles in increasing concentration of NaCl resulted in a steady decay of the swimming speed starting from a concentration *c*_NaCl_ = 1 × 10^–6^ M.^18^ Concentrations higher than *c*_NaCl_ = 1 × 10^–2^ M resulted in stuck colloids.

#### pH

**Figure 8:**
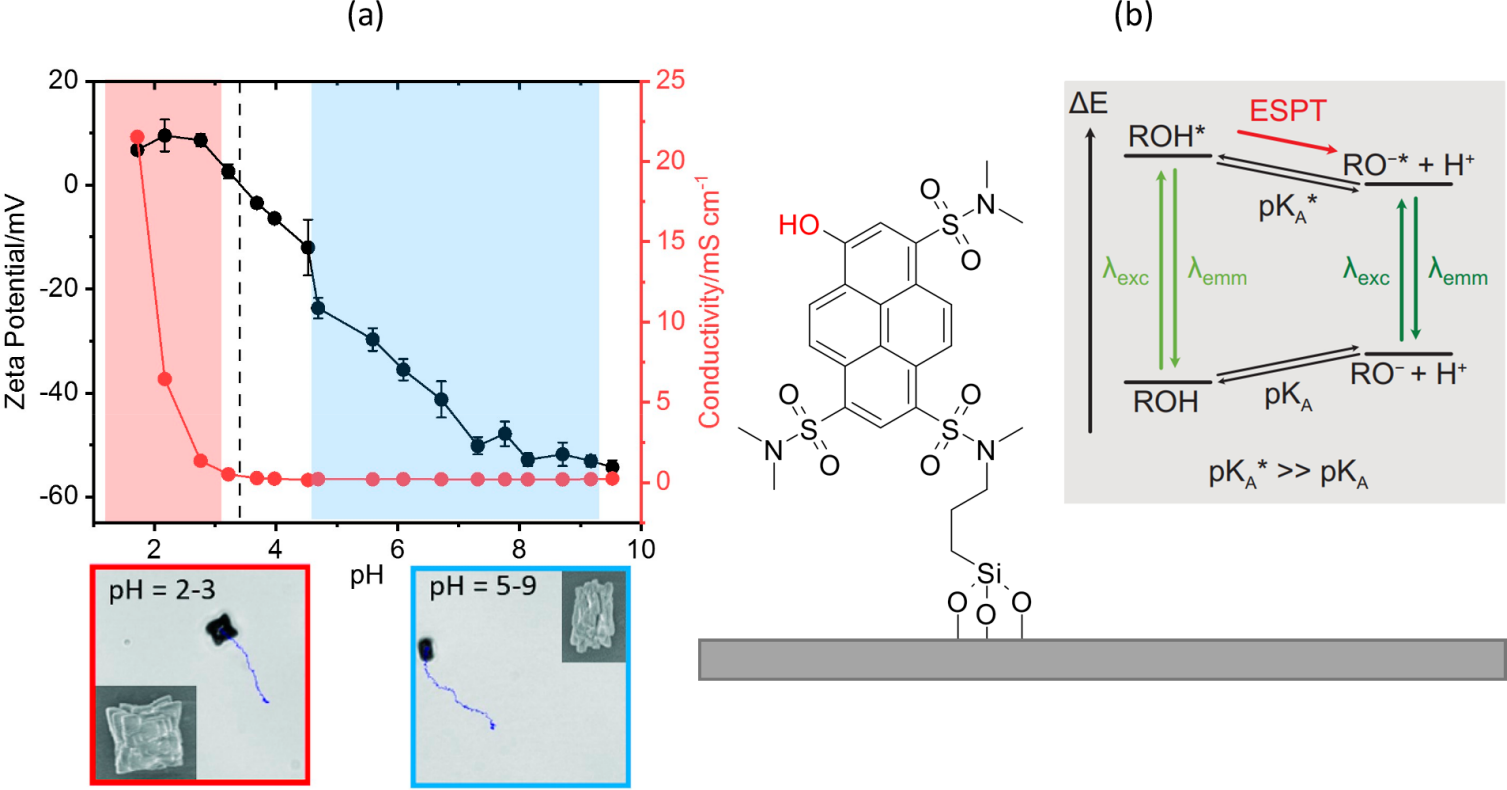
(a) Zeta potential and Conductivity of stacked BiVO_4_ particles at different pH values. Particles with positive zeta potentials perform horizontal motion (indicated by red area), while a reorientation and upright motion (indicated by blue area) was observed for negative zeta potentials. (b) Scheme showing immobilized HPDASCl on a glass substrate (left) and photoacid principle (right). Under UV illumination, an increased acid constant causes the dissociation of the molecule. Summarizing experiments from refs (18, 21). Copyright 2019 Wiley Online Library.

The motion of BiVO_4_ particles is strongly influenced by their surface charge, which depends on the pH value and the point-of-zero-charge. For stacked particles, we found that their motion mode changes from upright to horizontal depending on the pH values.^2,21^Figure 8a displays the zeta potential and conductivity of stacked BiVO_4_. From pH 5–9 the particles show a negative zeta potential, perform a reorientation and move in an upright orientation. However, if the pH is decreased and the zeta potential changes to positive values no reorientation and a horizontal motion is observed.

Based on this observation, we explored the possibility of locally influencing the pH in the vicinity of the particles. For that purpose, the photoacid 3,8-bis(*N*,*N*-dimethylsulfamoyl)-6-hydroxypyrene-1-sulfonyl chloride (HPDASCl) was coupled to APTES and immobilized on glass substrates.^16^ Under UV light irradiation, the p*K*_a_ value of HPDASCl in water decreases from 5.7 to −2.4, which causes a decrease on local pH and possibly inverts the zeta potential of the BiVO_4_ particles from negative to positive (see Figure 8b). Experiments were carried out in a dilute H_2_O_2_ solution at pH 6.2. Under these conditions, only upright motion was observed on unmodified glass substrates. However, on substrates with immobilized photoacid about 40%–80% of particles show upright motion and 12%–45% show horizontal motion. Additionally, we observed a third motion mode, that was called tumbling, where the particle either switches between horizontal and upright repeatedly or cannot be assigned to any of the two at all. On the other hand, the morphology of the microswimmers also affects their swimming behavior. A comparison between single and twin star shaped BiVO_4_ microswimmers showed that only the latter exhibited a stand-up motion upon light irradiation, attributed to a higher accumulation of the photogenerated products due to their concave morphology.

#### Chemical Fuels

**Figure 9:**
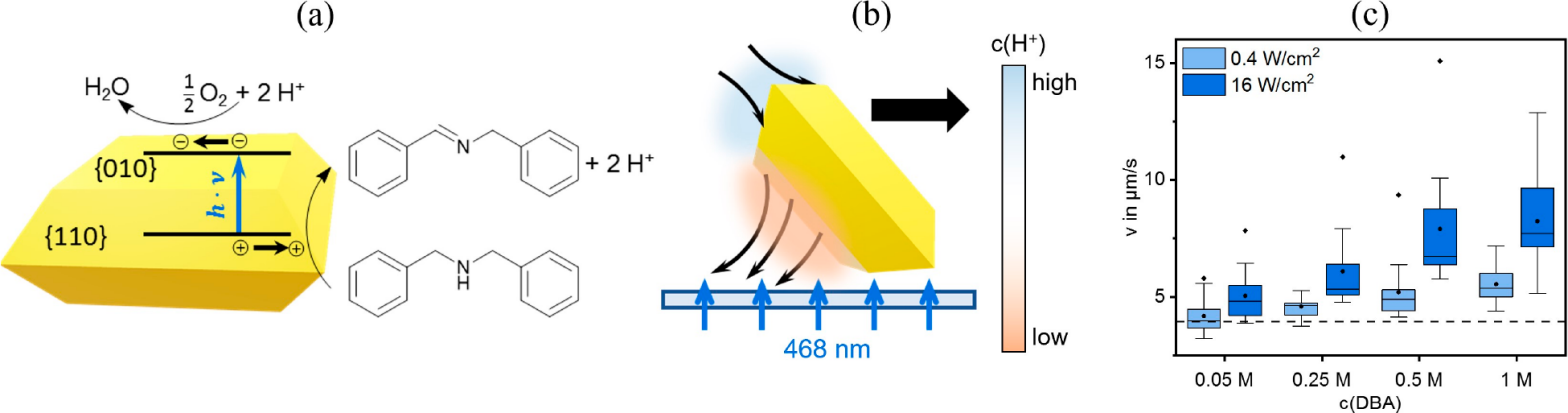
(a) Scheme showing the spatially separated half reactions on the facets of BiVO_4_ microcrystals. The oxidation of Dibenzylamine to *N*-Benzylidenebenzylamine is taking place on the {110} facets while O_2_ is oxidized to H_2_O on the {010} facets. (b) Scheme demonstrating the created gradients in H^+^ concentration and the resulting flows. (c) Box plots of average speeds of BiVO_4_ microswimmers under two intensities of blue light (468 nm) and in different DBA concentrations (*x* axis is not to scale). Reproduced with permission from ref (37). Copyright 2022 Wiley Online Library.

The previous studies in dilute H_2_O_2_ under UV illumination showed a dependence on fuel concentration and the influence of reaction- and catalyst-dependent equilibria.^18^ These experiments ensured the comparability with existing studies of Janus particle swimmers and theoretical models. However, the highly oxidative character of the photogenerated species upon activation of photocatalytic microswimmers can also be used to target other types of reactions, e.g. the oxidation of dibenzylamine (DBA) to *N*-benzylidenebenzylamine.^37^ Similar to the decomposition of H_2_O_2_, the oxidation reaction produces H^+^ while, at the same time, H^+^ ions are consumed in the reduction reaction (Figure 9a). Driven by the resulting flows, the particles moved in different concentrations of DBA in acetonitrile under visible light (Figure 9b and c), demonstrating that microswimmers can be propelled by an organic reaction in media different from those in aqueous solutions. Furthermore, we could also show, that nontraditional fuelling reactions, such as the photodeposition of metals from their respective salts, are suitable to propel BiVO_4_ micromotors.^38^ Noticeable therein is that because the gradient driving the motion is a negative one, the complex metal ions are removed from the solution and deposit on the BiVO_4_ surface.

## 5 Interactions

### 5.1 Active–Active Interactions

The formation of larger groups, so-called schools or flocks, often provides living active matter with remarkable functions, such as protection or foraging benefits. Often, these interactions happen without external coordination or supervision, resulting in a profound lack of understanding of group dynamics. So-called “arising of spontaneous complex phenomena” has fascinated physicists for decades, but we are still far away from understanding. Here, the interaction among seemingly simple, artificial active matter holds promises to abstract and model some of these interactions, which brings them to the attention of behavioral biologists as well as scientists developing future applications.

**Figure 10:**
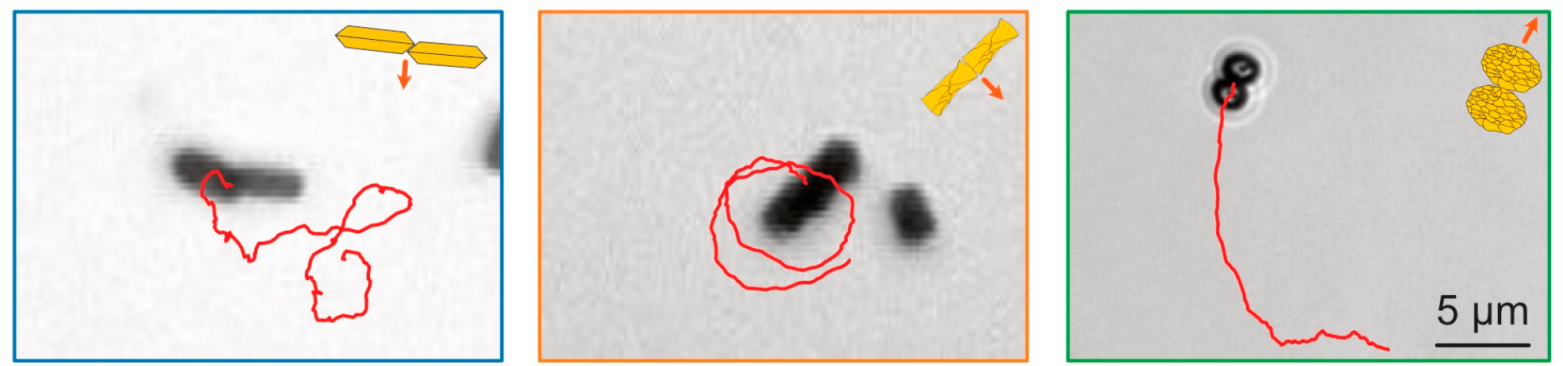
Light micrographs of assemblies of two BiVO_4_ microparticles of the same morphology, as guides to the eye, trajectories over several time units are represented with red lines. Single-crystalline and stacked particles assemble along their side faces and circular trajectories are observed. Spheroidal particles assemble along their short axis and do not show increased circularity in their trajectories. Comparing particle behaviors from refs (18, 20, 21, 23). Copyright held by the authors.

Modular microswimmers (i.e., reconfigurable assemblies of multiple particles into one larger swimming object) are a specific subspecies where a defined geometry may open possibilities to incorporate different functionalities into one swimmer. Generally, these assemblies are to be understood as an intermediate between single microparticles and swarms thereof.^39,40^ Previously, these mostly spherical building blocks were made of a single material without inherent structural anisotropy and therefore immotile when isolated, but upon assembly asymmetry is increased and motion is enabled.^39,40^ For BiVO_4_ microparticles, their inherently anisotropic semiconductor nature enables motility also in individual particles, but their nondipolar source geometry seems to favor attractive interactions. We observed attraction among all BiVO_4_ microparticle geometries: single-crystalline and stacked microparticles attract each other mainly along their side faces (Figure 10), which under illumination are hole-rich and seem to create mainly inward flows. As the particles are not permanently bound to each other, the assemblies reconfigure and break apart repeatedly under illumination. If the exciting UV light is turned off, all light-induced forces immediately vanish, which causes the particles to disassemble.

**Figure 11:**
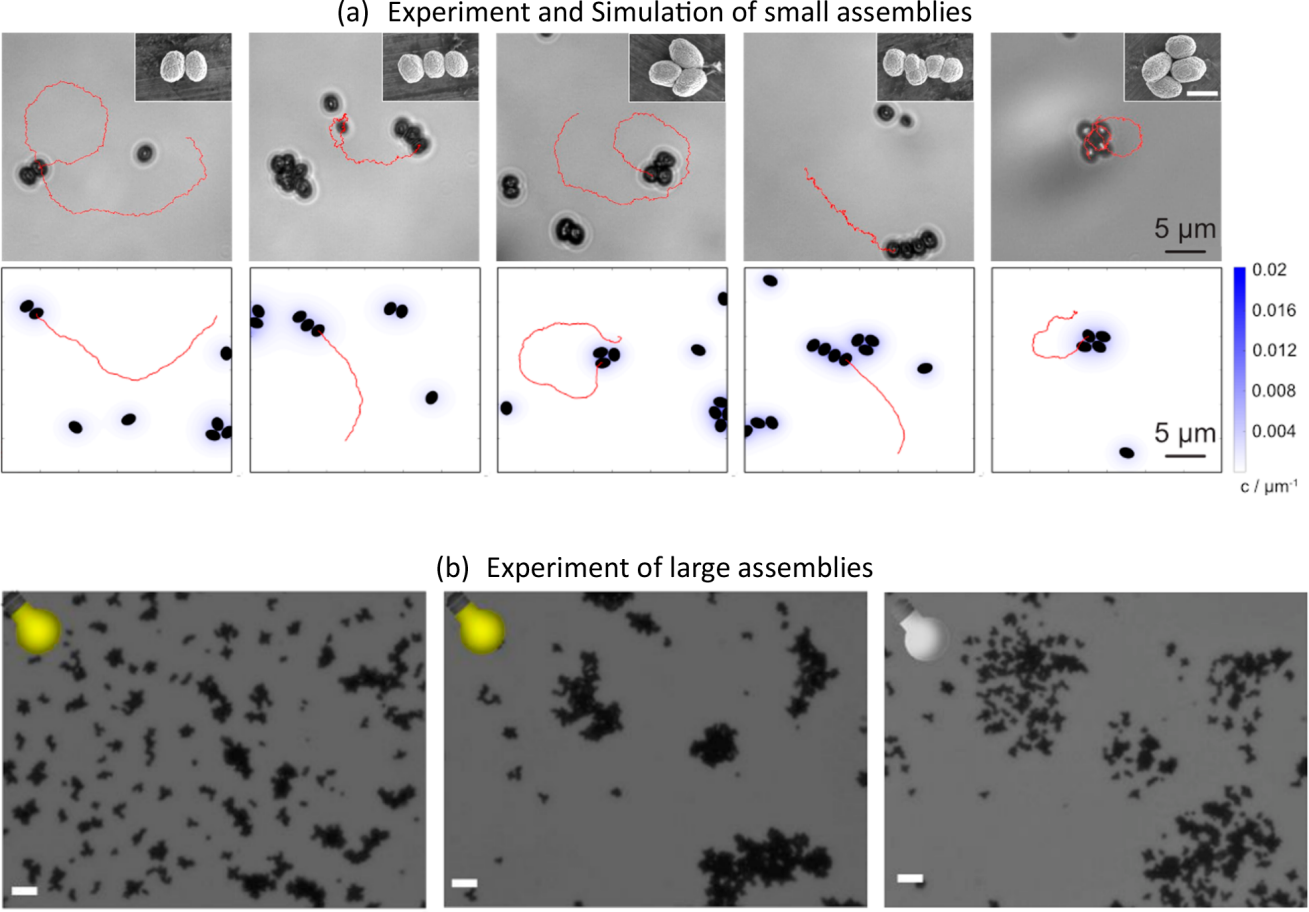
(a) Experimental micrographs (upper line) and simulation snapshots (lower line) of modular swimmers with recurring geometries consisting of two to four microparticles. Insets in experimental images show exemplary SEM images of the respective geometry for visualization. Inset scale bar is 2 μm. (b) Collective assemblies of star shaped BiVO_4_ micromotors induced by switching the light on/off. Scale bars: 10 um. Reproduced with permission from refs (20, 23). Copyright 2019 and 2020, respectively, American Chemical Society.

For spheroidal microswimmers, an increased tendency to form larger assemblies, preferably along their side faces, was observed. Upon contact with other spheroidal microparticles, these assemblies grow into motile, recurring geometries with up to four microparticles, that move actively (Figure 11a). Similarly, star shaped BiVO_4_ particles reversibly form larger clusters (Figure 11b).

For spheroidal microswimmers, the interactions were found to happen on the long side of the ellipsoids, i.e., the moving direction. To theoretically reproduce this behavior, interactions were implemented in solid particles, constructed from partially overlapping spheres with catalytic activity implemented along one side of the spheroids. In consequence, a chemical concentration field *c*(**r***,t*) is produced at a rate *k*_0_. All colloids in the system respond to this field, which effectively causes self-propulsion and attraction between colloids.

This model produces assemblies consisting of three and four microparticles in two different configurations: linear “caterpillars” and a more compact configuration, and reproduces the trends in speed. called “triangle” and “rectangle”. The speed of assemblies decreases with increasing amount of particles in experiments as well as in simulations, which is likely related to the change in the chemical gradient around larger assemblies in relation to their drag force. Toward a complete understanding of collective interactions of inherently anisotropic micromotors, including the larger, frequently immotile assemblies that were observed at long illumination times, further experiments are required.

### 5.2 From Active–Passive Interactions to Synergies with Microorganisms

Considering active matter in Nature, collective interactions are fascinating and highly relevant for certain systems such as bacteria within biofilms, while for other swimmers, such as sperm or algae, the probability to encounter obstacles or alien species is much higher.

Electrostatic and phoretic interactions are some of the most common causes inducing cargo transportation by photocatalytic microswimmers.^41^ Star-shaped BiVO_4_ micromotors have demonstrated a high driving force enabling the active particles to transport objects of different sizes, including 2 μm polystyrene (PS) particles, but also bigger objects that exceed the size of the micromotors. This type of micromotor can either rotate in place or show directional motion. It has been reported that, by rotating, they can easily grab/release micron-sized polystyrene particles by switching the light ON/OFF. The capabilities of BiVO_4_ micromotors to interact with living microorganisms were also investigated, but specific behaviors are discussed within the next section.

## 6 Applications

Photocatalytic systems can be used in a plethora of applications ranging from selective oxidation to reductive reactions. Ideally, smart micro/nanoswimmers should perform specific tasks in a programmable and autonomous way, i.e., without requiring constant human assistance for motion control. In Nature, similar mechanisms are known for living microorganisms, which are mediated by chemical signaling according to the environment conditions. Due to the ability to outswim diffusion, environmental remediation is one of the top applications for micromotors, but the scaling discrepancies between tiny colloids and huge problems are dramatic. Using larger collectives of microswimmers can be a way to solve this discrepancy. Self-propelled photocatalytic micromotors have been used mostly for degradation of organic pollutants and recently, for removing plastic-based pollutants.^42−44^ This oxidation reaction is mainly mediated by the generation of highly reactive species such as hydroxyl and superoxide radicals. In this section, we will discuss the main proof-of-concept applications of BiVO_4_ microswimmers that have been reported so far and offer a perspective on what might be required to push them beyond ’proof-of-concept’.

### 6.1 Degradation of Organic Pollutants

Similar to the majority of light-driven photocatalytic micromotors, BiVO_4_ has the ability to oxidize organic pollutants upon illumination. The coupling of BiVO_4_ with a second photoactive material is beneficial to enhance electron–hole pair separation, leading to higher photocatalytic activity than bare BiVO_4_. Dong et al. have reported a novel approach based on cooperative interactions between two types of micromotors, involving BiVO_4_ and BiVO_4_/graphene oxide micromotors with spherical and ellipsoidal morphology, respectively. The assembly/disassembly between micromotors can be easily controlled by adjusting the light intensity, due to an interplay between self-diffusiophoresis and electroosmotic mechanisms. Such cooperative interactions were used for the removal of rhodamine B as a model pollutant. The mixture of BiVO_4_ and BiVO_4_/graphene oxide micromotors achieved the highest pollutant removal efficiency in comparison with only BiVO_4_ micromotors. This has been attributed to the aggregation/disassembly among both types of motors under different light intensities that lead to a higher stirring of the liquid media, enhancing the mass transfer among RhB and the micromotors.^45^

### 6.2 Antimicrobial Activity

Besides chemical contamination in wastewater, the presence of pathogenic microorganisms is one of the major causes of waterborne infections associated with drinking-water safety. Photocatalytic-based microswimmers can act as pathogen-neutralizing devices, due to the *in situ* photogeneration of reactive oxygen species. Photocatalytic BiVO_4_ star-shaped swimmers are able to autonomously swim toward yeast cells (funghi), capture them by phoretic interactions and transport them under constant light irradiation.^20^ The surface of BiVO_4_ was not functionalized nor the swimming direction was manipulated by magnetic fields, indicating their autonomous behavior and intrinsic capabilities for capturing microorganisms. The BiVO_4_ microswimmers showed the same capability of capturing yeast cells in the presence of other type of microorganisms, such as *E. coli*. Although some bacteria could attach to the BiVO_4_ motors, it did not hamper their ability to swim toward yeast cells and attach to their surface. Even though this behavior has been ascribed to a combination of several factors, including roughness surface, hydrophobicity, and surface interactions, the exact mechanisms of such interactions are not well understood. Before any real applications can be tackled, this lack of understanding needs to be addressed.

### 6.3 Food Industry

**Figure 12:**
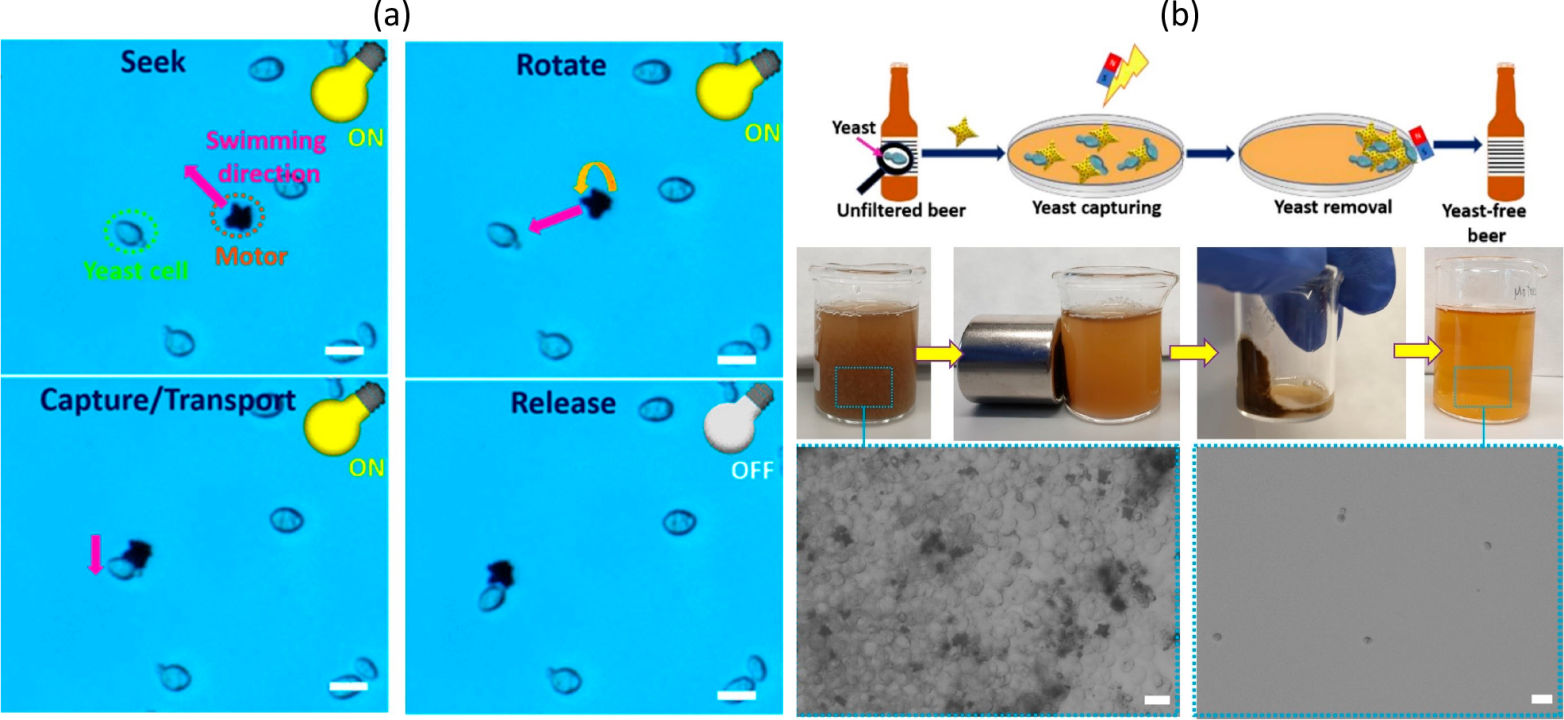
(a) Intrinsic interactions of BiVO_4_ microswimmers with yeast cells under visible light irradiation, involving a swimming trajectory change to capture and transport a yeast cell. (b) Schematic illustration of yeast cell removal by BiVO_4_/Fe_3_O_4_ microswimmers from an unfiltered beer under combined light/magnetic fields. It also shows an optical microscope image of a beer sample before and after treatment. Reproduced with permission from refs (20, 46). Copyright 2019 American Chemical Society and 2020 Chemistry Europe, respectively.

Yeast plays a key role in the manufacturing of food and alcoholic beverages. Therefore, the spontaneous interactions between artificial microswimmers and yeast cells can have a plethora of applications. Common approaches for yeast removal after the fermentation processes rely on pasteurization or filtration treatments. BiVO_4_ star-shaped microswimmers modified with magnetic nanoparticles were shown to remove yeast contamination in real samples, such as alcoholic beverages (see Figure 12) because the self-propelled BiVO_4_ microswimmers autonomously bind to yeast cells. Owing to their dual optical/magnetic response, the resulting assembly micromotor/yeast can be easily removed after treatment, simplifying the whole removal process. The treatment of unfiltered beer with BiVO_4_/Fe_3_O_4_ micromotors resulted in almost 100% yeast removal. More importantly, there were no differences in the organoleptic properties of beer before and after treatment. The leakage of Fe and V ions was also examined, finding only remaining traces that are allowed in drinking water. Therefore, this work represents a simple approach to overcoming yeast spoilage in alcoholic beverages for applications in microbreweries that can be extended to other food-related processes.

There are diverse applications for BiVO_4_ micro/nanomotors that are yet to be explored. For instance, BiVO_4_ is a well-known yellow pigment with antifouling properties.^47^ Moreover, it has been widely explored for photoelectrochemical water oxidation, mainly decorated with cocatalysts or a second semiconductor. On the other hand, owing to the mild oxidizing power in comparison to TiO_2_, it is also a very interesting material for the generation of liquid solar fuels from methane, such as methanol.^48^ Therefore, the improvement of the micromixing by the active self-propulsion of BiVO_4_ micromotors might result in superior efficiencies of such challenging selective oxidations or add new dimensions for previous well-established reactions. Another interesting approach involves their coupling with enzymatic components for potentially driving photobiocatalytic reactions or enhancing and controlling the motion and functionalities of enzyme-based micro/nanoswimmers.^49,50^

## 7 Conclusions and Outlook

In this Account, we discuss the importance and versatility of BiVO_4_ not only as a material for photoelectrochemical processes but also as an efficient photocatalyst for developing efficient light-driven microswimmers. Owing to its comparatively small bandgap, BiVO_4_ microswimmers can propel under visible light irradiation, which is desirable for a variety of applications that otherwise are hampered by the use of harmful UV wavelengths. Therefore, this feature enables the application of BiVO_4_ microswimmers in water disinfection under biocompatible light wavelenghts.

Besides, BiVO_4_ can be synthesized in multiple shapes by easily scalable techniques, mainly involving co-precipitation and hydrothermal treatments. Depending on the morphology, from spherical, star-shaped, to single-crystals, BiVO_4_ microswimmers show interesting swimming behaviors. For instance star-shaped micromotors show an unique stand-up motion behavior, due to chemical gradient build-up between the particle and the substrate. On the other hand, single-crystal-based micromotors show enhanced speeds in comparison with polycrystalline ones, which is atributed to an spatial electron–hole separation of the photogenerated excitons in each crystal facet. Therefore, unlike common Janus microswimmers based on metal–semiconductor heterojunctions, BiVO_4_ based devices can effectively separate electron–hole pairs without requiring the inclusion of an additional component in the micromotor structure. In this way, we can skip the use of large and expensive equipment, such as metal evaporators.

Their motion is affected by the surrounding pH, properties of the substrate, and type of fuel, offering more options for their actuation and manipulation. BiVO_4_ microswimmers have also the ability to interact with each other and with the surroundings, which has led to emerging collective interactions, such as clustering-like behaviors and/or self-assembly, which are common for polycrystalline spheroidal particles. Such interactions have been useful to develop different microswimmer-based configurations and perform cargo transportation of passive particles (e.g., polystyrene) and living microorganisms. When yeast cells were selected as model microorganisms, the polycrystalline stacked BiVO_4_ swimmers moved toward the yeast and captured it, involving even change of their swimming direction. However, further studies including flow field analysis are required to fully understand such interactions, which will enable the development of more sophisticated and controllable photoactive microdevices for biomedical applications such as photodynamic therapy. The low price and toxicity associated with this material raise expectations for applications beyond proof-of-concept for this convenient material. However, more systematic studies dealing with the reusability of these micromotors in multiple tests and an investigation of possible leaching of metals upon irradiation should be considered in the future. Moreover, the decoration of BiVO_4_ with other semiconductors and/or materials would be beneficial to induce their motion in H_2_O_2_-free environments to avoid adding extra pollution in the liquid media.

## Supporting Information Available

Movie showing single crystalline, polycrystalline stacked, and polycrystalline spheroidal particles (AVI)
Movie showing yeast capture by a particle (MP4)
Synthesis and characterization of BiVO_4_ particles (PDF)

## Supplementary Material

mr3c00021_si_001.avi
