# Supplementary material for: An Account on BiVO4 as Photocatalytic Active Matter
Source: Acc Mater Res. 2024 Mar 15;5(4):400–12. doi: 10.1021/accountsmr.3c00021 (PMC11059100; doi:10.1021/accountsmr.3c00021)
Supplement: Supplementary file 3 — mr3c00021_si_003.pdf [file mr3c00021_si_003.pdf]

An account on $BiVO_4$ as photocatalytic active matter

Sandra Heckel,[†] Martin Wittmann,[†] Marc Reid,[‡] Katherine Villa,[*,¶] and Juliane Simmchen[*,†]

†*Physical Chemistry, TU Dresden, Zellescher Weg 19, 01069 Dresden, Germany*

‡*Department of Pure and Applied Chemistry, University of Strathclyde, 295 Cathedral Street, Glasgow G1 1XL*

¶*Institute of Chemical Research of Catalonia (ICIQ), The Barcelona Institute of Science and Technology (BIST), Av. Països Catalans, 16,43007, Tarragona, Spain*

E-mail: kvilla@iciq.es; juliane.simmchen@tu-dresden.de

Experimental Details

Synthesis of $BiVO_4$ particles

The synthesis of **spheroidal and stacked polycrystalline $BiVO_4$ particles** was carried out according to[1,2] and is based on a protocol reported by Jiang et al. with some modifications.[3] 12.5 mL of a 1:1 mixture of ethanol/ethylene glycol mixture were prepared in a 100 mL Erlenmeyer flask and 2.5 mL of concentrated HNO_3 and 2.78 g (15 mmol) of dodecylamine were added under stirring. After that, 2.425 g (5 mmol) $Bi(NO_3)_3 \cdot 5\,H_2O$ and 0.585 g (5 mmol) NH_4VO_3 were added and dissolved. After the salts are dissolved, the pH was adjusted using a 2 M NaOH in a 1:1 ethanol/ethylene glycol mixture. Adjusting to pH 2 lead to particles with spheroidal morphology and stacked particles were obtained at pH 3.

The solution was transferred to a Teflon-lined stainless steel autoclave and left to ripen for 3 h, followed by a hydrothermal treatment at 100 °C for 12 h. After centrifugation and washing with ethanol and water, the sample was dried at 60 °C for 12 h and stored under air.

The synthesis of **single-crystalline $BiVO_4$ particles** was carried out by a hydrothermal reaction adapting a procedure by Li et al..[4,5] $Bi(NO_3)_3 \cdot 5\,H_2O$ (5 mmol, 2.425 g) was dissolved in 20 mL 2 M HNO_3. After that, NH_4VO_3 (5 mmol, 0.585 g) was added under stirring leading to a yellow solution. The pH was adjusted to pH 2 using NH_3 (16 % in H_2O). NaCl was added to a final concentration of 0.05 M and sodium dodecyl sulfate to a concentration of 0.005 M. After 15 min of stirring, the solution was transferred to a Teflon-lined stainless steel autoclave and left to ripen for 2 h, followed by a hydrothermal treatment at 200 °C for 24 h. After centrifugation and washing with water, the sample was dried at 60 °C for 12 h and stored under air.

Characterization

Absorption spectra were measured in a Cary 60 UV-Vis spectrophotometer from Agilent Technologies. Samples were either prepared as diluted aqueous dispersions or monolayer films on glass substrates. Samples were generally measured in an integrating sphere to account for scattering on the particle surface. Additionally absorption and transmission of films was measured in a direct beam path used for conventional measurements.

XRD analysis was done on a Bruker 2D phaser in a 2θ range between 10 and 100°.

Dye degradation experiments were carried out using rhodamin B. A stock solution of rhodamin B with a concentration of $10\,\mathrm{mg\,l^{-1}}$ ($2 \times 10^{-2}\,\mathrm{mmol\,l^{-1}}$) as well as microparticle dispersions with a concentration of $15\,\mathrm{g\,l^{-1}}$ were prepared. In a quarz cuvette with 1 cm path length, 100 µl of particle dispersion were added to 3 ml rhodamin B solution. After thorough mixing by ultrasonication, the solution was stirred in the dark for 20 min, after which an absorption spectrum was taken. The cuvette was then illuminated by a 220 V high-power

halogen lamp under stirring for 4 h. Absorption spectra were taken after 30 min, 1 h, 2 h, 3 h and 4 h. All particle morphologies were illuminated in a separate quarz cuvette at the same time.

Characterization

Absorption spectra and corresponding Tauc plots are shown in Figure 1. All particle morphologies display the typical absorption spectrum of a bulk semiconductor and absorb light in the UV up to the visible light range.

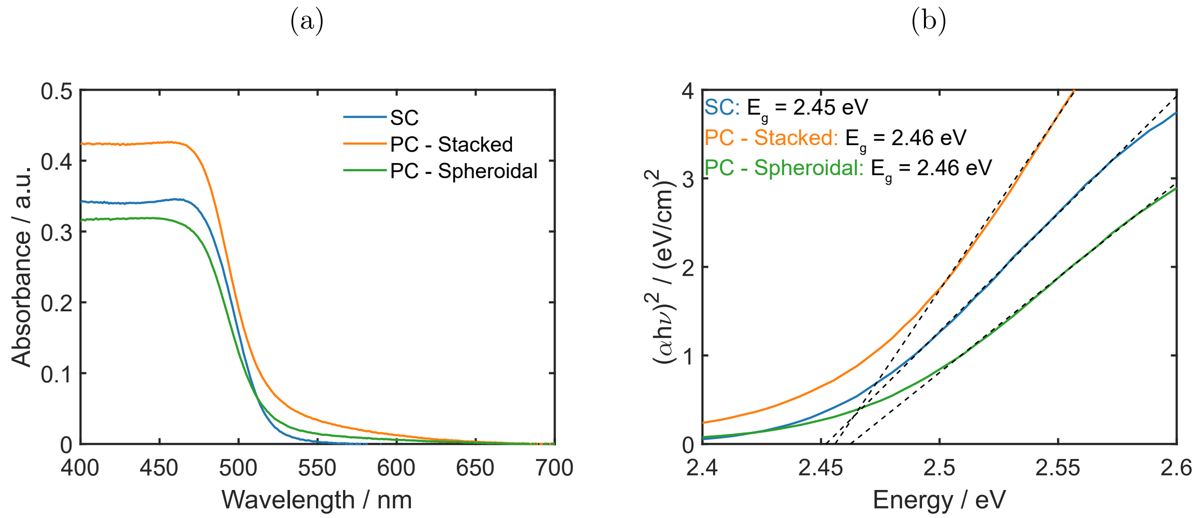

Figure 1: (a) Absorption spectra and (b) Tauc plots of single-crystalline, stacked and spheroidal polycrystalline $BiVO_4$ microparticles. For the single-crystals, the band gap energy is 2.45 eV, for both polycrystalline particles 2.46 eV, i.e. all particle morphologies can be excited with light up to the blue range of the visible light spectrum to create excited charge carriers. Reproduced with permission from reference.[6]

Comparing the photocatalytic activity of the different particles in a dye degradation experiment with rhodamin B reveals the highest activity for the single-crystalline particles followed by the stacked and spheroidal polycrystalline particles. XRD analysis confirms the monoclinic $BiVO_4$ structure, where the larger crystallite size in the single-crystals causes sharper peaks. The stacked particles also contain small amounts of tetragonal $BiVO_4$ phase.

(a) (b)

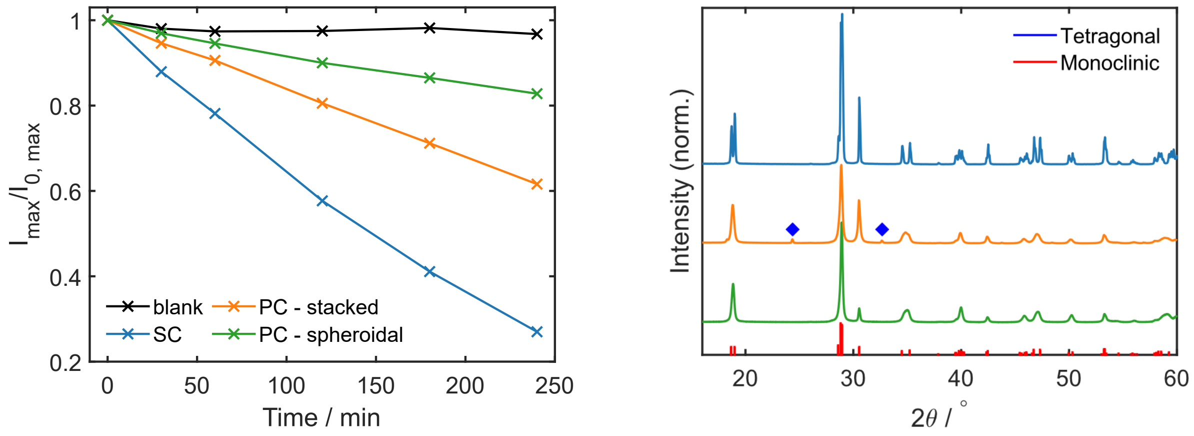

Figure 2: (a) Relative intensity of the absorption maximum of rhodamine B over time when photocatalytically decomposed by single-crystalline (SC) and polycrystalline (PC) $BiVO_4$ particles. (b) XRD of these particles (same color code as in (a)). Reproduced with permission from reference.[6]

Further reading

- General review on $BiVO_4$ and improvement of photocatalytic activity[7–9]
- Band edges and electronic structure,[10] specific for monoclinic $BiVO_4$[11,12]
- Charge carrier separation on the different facets of $BiVO_4$[13,14] and other photocatalysts[15]
- Other morphologies[7]
- Nanoscale $BiVO_4$[16,17]

References

(1) Heckel, S.; Simmchen, J. Photocatalytic BiVO4 Microswimmers with Bimodal Swimming Strategies. *Advanced Intelligent Systems* **2019**, *1*, 1900093.

(2) Heckel, S.; Grauer, J.; Semmler, M.; Gemming, T.; Löwen, H.; Liebchen, B.; Simmchen, J. Active assembly of spheroidal photocatalytic BiVO4 microswimmers. *Langmuir* **2020**, *36*, 12473–12480.

(3) Jiang, H.; Dai, H.; Meng, X.; Ji, K.; Zhang, L.; Deng, J. Porous olive-like BiVO4: Alcoho-hydrothermal preparation and excellent visible-light-driven photocatalytic performance for the degradation of phenol. *Applied Catalysis B: Environmental* **2011**, *105*, 326–334.

(4) Li, R.; Zhang, F.; Wang, D.; Yang, J.; Li, M.; Zhu, J.; Zhou, X.; Han, H.; Li, C. Spatial separation of photogenerated electrons and holes among {010} and {110} crystal facets of BiVO 4. *Nature Communications* **2013**, *4*, 1432–1437.

(5) Heckel, S.; Bilsing, C.; Wittmann, M.; Gemming, T.; Büttner, L.; Czarske, J.; Simmchen, J. Beyond Janus Geometry: Characterization of Flow Fields around Nonspherical Photocatalytic Microswimmers. *Advanced Science* **2022**, *n/a*, 2105009.

(6) Heckel, S. Inherently Asymmetric Photocatalytic Microswimmers. Ph.D. thesis, TU Dresden, Dresden, Germany, 2021.

(7) A., M.; J., M.; Ashokkumar, M.; Arunachalam, P. A review on BiVO 4 photocatalyst: Activity enhancement methods for solar photocatalytic applications. *Applied Catalysis A: General* **2018**, *555*, 47–74.

(8) Liu, X.; Gu, S.; Zhao, Y.; Zhou, G.; Li, W. BiVO4, Bi2WO6 and Bi2MoO6 photocatalysis: A brief review. *Journal of Materials Science Technology* **2020**, *56*, 45–68, Solar-driven Photocatalystic Materials.

(9) Lotfi, S.; Ouardi, M. E.; Ahsaine, H. A.; Assani, A. Recent progress on the synthesis, morphology and photocatalytic dye degradation of BiVO4 photocatalysts: A review. *Catalysis Reviews* **2022**, *0*, 1–45.

(10) Walsh, A.; Yan, Y.; Huda, M. N.; Al-Jassim, M. M.; Wei, S.-H. Band edge electronic structure of BiVO4: elucidating the role of the Bi s and V d orbitals. *Chemistry of Materials* **2009**, *21*, 547–551.

(11) Zhao, Z.; Li, Z.; Zou, Z. Electronic structure and optical properties of monoclinic clinobisvanite BiVO 4. *Physical Chemistry Chemical Physics* **2011**, *13*, 4746–4753.

(12) Cooper, J. K.; Gul, S.; Toma, F. M.; Chen, L.; Glans, P.-A.; Guo, J.; Ager, J. W.; Yano, J.; Sharp, I. D. Electronic structure of monoclinic BiVO4. *Chemistry of Materials* **2014**, *26*, 5365–5373.

(13) Lardhi, S.; Cavallo, L.; Harb, M. Significant Impact of Exposed Facets on the BiVO4Material Performance for Photocatalytic Water Splitting Reactions. *Journal of Physical Chemistry Letters* **2020**, *11*, 5497–5503.

(14) Liu, T.; Zhou, X.; Dupuis, M.; Li, C. The nature of photogenerated charge separation among different crystal facets of BiVO4 studied by density functional theory. *Phys. Chem. Chem. Phys.* **2015**, *17*, 23503–23510.

(15) Guan, L.; Chen, X. Photoexcited Charge Transport and Accumulation in Anatase TiO2. *ACS Applied Energy Materials* **2018**, *1*, 4313–4320.

(16) Sivakumar, V.; Suresh, R.; Giribabu, K.; Narayanan, V. BiVO4 nanoparticles: Preparation, characterization and photocatalytic activity. *Cogent Chemistry* **2015**, *1*, 1074647.

(17) Sun, W.; Xie, M.; Jing, L.; Luan, Y.; Fu, H. Synthesis of large surface area nano-sized BiVO4 by an EDTA-modified hydrothermal process and its enhanced visible photocatalytic activity. *Journal of Solid State Chemistry* **2011**, *184*, 3050–3054.
